# Acute liver failure due to visceral leishmaniasis in Barcelona: a case report

**DOI:** 10.1186/s12879-019-4553-7

**Published:** 2019-10-22

**Authors:** Iratxe Martinez de Narvajas, Alba Díaz, Octavio Bassegoda, Adriá Carpio, Carla Fuster, María Eugenia Valls, Miriam J. Alvarez-Martínez, Carolina García-Vidal, Alejandro Soriano, José Antonio Martínez, Juan Ambrosioni

**Affiliations:** 1 Internal Medicine Service, Hospital San Pedro, Logroño, Spain; 2 Infectious Diseases Service Hospital Clinic-IDIBAPS, Villarroel 170, 08032 Barcelona, Spain; 3 Pathology Service, Hospital Clinic-IDIBAPS, Barcelona, Spain; 4 Hepathology Service, Hospital Clinic-IDIBAPS, Barcelona, Spain; 5 Microbiology Dpt-CDB, Hospital Clinic-ISGLOBAL, Barcelona, Spain

**Keywords:** Visceral leishmaniasis, Liver failure, *Leishmania*, *Leishmania infantum*

## Abstract

**Background:**

Leishmaniasis is an emerging infectious disease. Due to human migration and tourism, visceral leishmaniasis may become more common in non-endemic areas. In the Mediterranean basin, visceral leishmaniasis typically occurs in rural regions.

**Case presentation:**

We present an unusual urban case of acute liver failure due to visceral leishmaniasis, following a prolonged fever of unknown origin. After obtaining negative results from the bone marrow aspirate, we performed a liver biopsy that elucidated the diagnosis. The liver involvement in visceral leishmaniasis may appear as chronic granulomatous hepatitis. However diffuse hepatitis process, a necro-inflammatory pattern, without forming granulomas were observed in the liver biopsy specimens in this case. Intracytoplasmic *Leishmania* amastigotes were observed in the liver biopsy specimens and a polymerase chain reaction confirmed the diagnosis. Only five pathological confirmed cases of acute hepatitis due to visceral leishmaniasis have been described so far, just two in adults and both from Barcelona. A revision of the literature is performed.

**Conclusions:**

Acute hepatitis is an uncommon debut of visceral leishmaniasis in immunocompetent patients. Furthermore there are only few cases in the literature that describe the histopathological changes that we found in this patient. In conclusion, in case of acute hepatitis leading to liver failure, leishmaniasis should be considered a differential diagnosis (even in non-endemic countries and without clear epidemiological exposure) and liver biopsy can elucidate the diagnosis.

## Background

Leishmaniasis is an emerging infectious disease, caused by protozoa that live inside macrophages in mammals. Sandflies become infected after feeding on the reservoir animal (rodents, dogs, and other small mammals) or infected humans and can then transmit the parasite to other humans [1].

Clinical manifestations are dependent both on the infecting species of *Leishmania* and the immune response of the host. Many cases are asymptomatic, reflecting the ability of the host immune system to control the parasite. There are two main clinical forms: visceral leishmaniasis and cutaneous leishmaniasis. Cutaneous leishmaniasis presents as single or multiple papular, nodular or ulcerous skin lesions. Visceral leishmaniasis is the most serious clinical form of this disease. Persistent fever, splenomegaly and pancytopenia characterize visceral leishmaniasis [1]. An infrequent form of leishmaniasis is localized leishmanial lymphadenopathy without fever or skin lesions. After the largest outbreak of leishmaniasis in Europe a series of cases of localized leishmanial lymphadenopathy was published. The presentation as fulminant hepatitis is even more uncommon [2].

The global incidence of visceral leishmaniasis decreased substantially in the past decade: from between 200,000 and 400,000 new cases in 2012, to between 50,000 and 90,000 new cases in 2017 [1]. Among tropical diseases, leishmaniasis ranks second in mortality and seventh in loss of disability-adjusted life years [3]. Outbreaks of human leishmaniasis are unusual in Spain, despite being considered an endemic disease. Here we describe a case of acute hepatitis with liver failure as visceral leishmaniasis presentation in an immunocompetent adult from an urban area in Barcelona.

## Case presentation

A 55-year-old man resident in Barcelona was evaluated for a 2-week history of unexplained fever despite antipyretic treatment. Medical record was positive only for hypertension controlled with enalapril. He was born in Barcelona and he always lived there. 2 years ago he had traveled to China and Israel, and 10 years ago to several countries of South America, with no other relevant epidemiological risk. The patient denied alcohol intake previous to admission At admission he was 39.1 °C, the heart rate was 89 bpm, and the blood pressure was 123/66 mmHg. Physical examination was unremarkable. Initial blood test revealed increased C-reactive protein levels to 10.55 mg/dL, elevated aspartate aminotransferase (88 U/L), alanine aminotransferase (76 U/L), alkaline phosphatase (202 U/L) and gamma glutamyl transferase (269 U/L). Bilirubin and prothrombin time were within normal range. The hemoglobin was 12.7 g/dl and laboratory test showed leukopenia (2.83 × 10^9^/L) and thrombopenia (115 × 10^9^). Blood and urine cultures were taken on admission. Chest radiography was normal. Due to fever and analytical results it was decided to perform an abdominal ultrasonography revealing a hypoechoic lesion suggestive of a subcapsular splenic infarction. Infective endocarditis was suspected, starting an antibiotic combination of intravenous ampicillin 2 g/ 4 hs, cloxacillin 2 g/4 hs and ceftriaxone 2 g/12 hs. Transthoracic echocardiography was unremarkable and blood cultures were negative, discontinuing ampicillin and cloxacillin.

The patient continued having febrile peaks over 2 weeks, treated mainly by physical measures to avoid hepatotoxic drugs. A computed tomography revealed 15 cm-splenomegaly and several splenic infarctions, with no other abnormalities. Repeated blood analysis showed worsening of liver tests (AST 1649 U/L, ALT 911 U/L, gamma GT 447 U/L, total bilirubin 4.20 mg/dl, ammonium 82 μmol/L, albumin 2.4 mg/dl, prothrombin time of 40%), pancytopenia (hemoglobin 8 g/L, platelets 48 × 10^9^), high levels of triglycerides 222 mg/dl, elevated ferritin 12.886 ng/mL, lactic dehydrogenase 915 U/L, total proteins 55 g/l, normal kidney function test and normal blood smear.

**Fig. 1:**
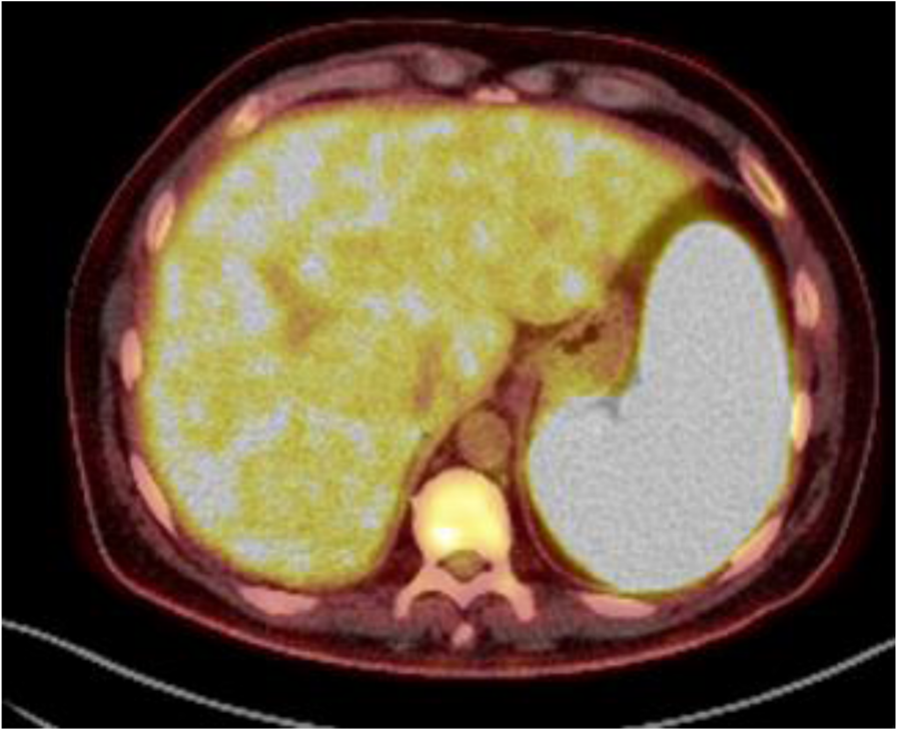
FDG-PET/TC. FDG-PET scans shows diffuse high uptake in the spleen (SUV máx: 13.26), liver, and bone marrow

Microbiology investigations (including serologies and molecular biology) were negative for tuberculosis, *Coxiella*, *Brucella*, Human immunodeficiency virus, viral hepatitis (A to E), *Cytomegalovirus*, Epstein-Barr virus and parvovirus B19. IgG for *Leishmania* was positive, with a title of 1:200 by IIFT technique and IgM was negative. He had low titers of antinuclear and anti-smooth muscle antibodies (1:80). Given these findings it was decided to perform a bone marrow aspiration and a ^18^F-fluorodeoxyglucose-positron emission tomography in combination with computed tomography scanning (FDG-PET/TC). There were no signs of haematologic malignancy in the bone marrow and there were no hemophagocytosis. Aspirated material inoculated into parasitic growth media and a Giemsa-stained smear was made, both were negative. FDG-PET/TC revealed a diffuse high uptake in the spleen, liver, and bone marrow but without involvement of lymph nodes or other organs (Fig. 1).

**Fig. 2:**
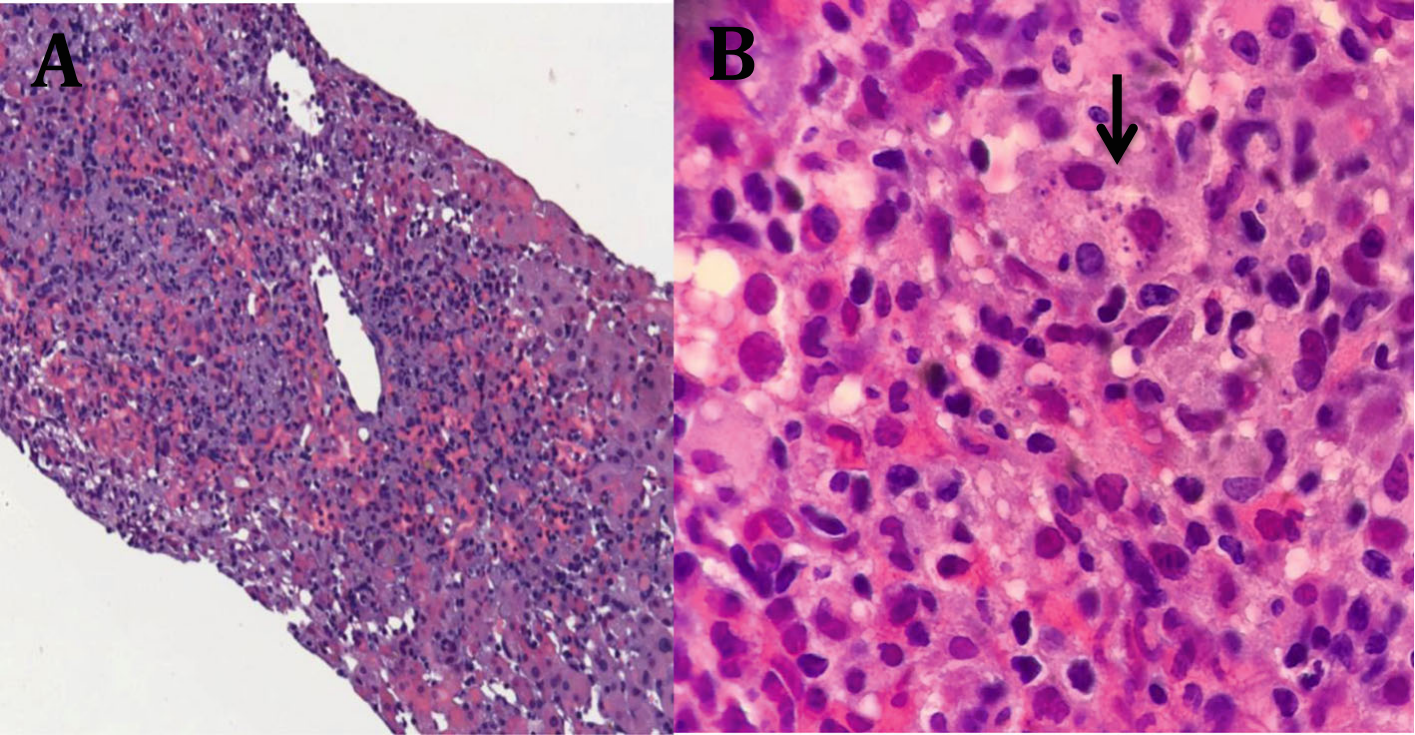
Histopathological study of the liver. **a** On the left side, liver biopsy showed an acute hepatitis with centrilobular necrosis, mixed inflammation and abundant Kupffer cells (Hematoxilin & Eosin, 10X). **b** Scattered small oval structures with rod-shaped kinetoplast (black arrow) were observed in the cytoplasm of some hepatocytes and Kupffer cells (Hematoxilin & Eosin, 40X)

Given the worsening of liver tests, a diagnostic liver biopsy was performed and histopathology showed an acute hepatitis with confluent necrosis and mixed inflammatory infiltrates with abundant plasmatic and histiocytic cells. Isolated macrophages with intracytoplasmic *Leishmania* amastigotes were observed (Fig. 2). Polymerase chain reaction in tissue was positive for *Leishmania infantum*. The patient started treatment with liposomal amphotericin B 3 mg/kg/day for 5 days and two additional single weekly doses, at day 14th and 21st, showing slow but continuous analytical and clinical involvement. He was completely recovered 1 month later.

## Discussion and conclusion

In 2017 the global incidence of visceral leishmaniasis was 50.000–90.000 new cases. Persistent irregular fever, splenomegaly, pancytopenia, weight loss and hypergammaglobulinemia characterize visceral leishmaniasis. Hepatomegaly is common in patients with visceral leishmaniasis and late in the course of disease, hepatic dysfunction, jaundice, and ascites can occur [1]. However rapid acute progressive hepatitis as a presenting feature is rare.

Definitive diagnosis of visceral leishmaniasis requires demonstration of the parasite by either histopathology or culture of material obtained by needle aspiration or biopsy from affected organs (usually bone marrow or spleen). In our case we performed a bone marrow aspiration and aspirated material inoculated into parasitic growth media and a Giemsa-stained smear was made, but both were negative. Unfortunately, PCR was not available in our institution, what may have provided an earlier diagnosis. Death occurs in more than 90% of patients without specific anti-leishmanial treatment. Therefore, a correct diagnosis of visceral leishmaniasis is essential. After obtaining negative results from the bone marrow aspirate, we performed a liver biopsy. Intracytoplasmic *Leishmania* amastigotes were observed in the liver biopsy specimens and a polymerase chain reaction requested to the national reference center confirmed the diagnosis.

Only few cases of acute hepatitis as a presenting form of visceral leishmaniasis are reported in the literature and many of them do not have a histopathological study of the liver. Just 4 previous cases with a liver biopsy were reported so far. The cases are summarized in the table (Table 1) [4–7]. Three of the published cases are in children, in Indian [7]. Since visceral leishmaniasis preferentially affects children or immunosuppressed adults, interestingly, in this adult patient, no evidenced of immunodeficiency was detected.

**Table 1:** Summary of patients with acute hepatitis due to visceral leishmaniasis and histopathology confirmation with liver biopsy.

	Age	Gender	Region	Jaundice	Hepatomegaly	Splenomegaly	AST (U/L)	ALT (U/L)	Alkaline phosph. (U/L)	Gamma GT (U/L)	Bilirrubin (mg/dl)	PT (sec)	Bone marrow	Liver biopsy	Outcome
Index case	55	Male	Barcelona Spain	No	No	Yes	1649	911	1107	447	12	15,6	Reactive changes, no infection	Necroinflammatory changes. Amastigotes	Alive
Bassegoda et al [6]	48	Male	Barcelona Spain	Yes	Yes	Yes	1326	645	407	545	5	15,2	Reactive changes, no infection	Necroinflammatory changes. Amastigotes	Alive
Singh et al I [7]	3	Male	Bihar India	NR	Yes	NR	700	800	NR	NR	NR	22	Amastigotes	Necrosis, granuloma.	Death
Singh et al II [7]	5	Male	Bihar India	NR	NR	NR	550	450	NR	NR	NR	18	Amastigotes	Necrosis, granuloma.	Alive
Singh et al III [7]	1	Female	Bihar India	NR	NR	NR	800	650	NR	NR	NR	20	Amastigotes	Necrosis, granuloma.	Death

*AST* Aspartate aminotransferase, *ALT* Alanine aminotransferase, *Phosph* Phosphatase, *GT* Gamma glutamyl transferase, *PT* Prothrombin time, *NR* Not reported

The liver involvement in visceral leishmaniasis may appear as chronic granulomatous hepatitis [8]. *El Hag* et al has reported histopathology changes in the liver in visceral leishmaniasis [9]. All cases had inflammation, Kupffer cell hyperplasia, intracellular amastigotes, focal and piecemeal necrosis. The histopathological findings of the case reported here mimics that published last year by Bassegoda et al. (also from Barcelona) and evidenced diffuse hepatitis process, a necro-inflammatory pattern, accompanying inflammatory infiltrate was mixed, with abundant plasmatic and histiocytic cells, without forming granulomas [6]. Very small, oval structures in the cytoplasm of some macrophages were suggestive of *Leishmania* amastigotes. The disease is rare in Spain and no common exposure or link could be traced between the two cases. Although a clear epidemiological exposure for our case was not identified, *Leishmania infantum* is endemic in the Mediterranean basin and therefore, exposure could have occurred even in Spain [1]. Interestingly the two only adult cases were from Barcelona and both had negative *Leishmania* detection in bone marrow samples.

Acute hepatitis is an uncommon debut of visceral leishmaniasis in immunocompetent patients. Furthermore there are only few cases in the literature that describe the histopathological changes that we found in this patient. In conclusion, in case of acute hepatitis leading to liver failure, leishmaniasis should be considered a differential diagnosis (even in non-endemic countries and without clear epidemiological exposure) and liver biopsy can elucidate the diagnosis.

## Data Availability

Data sharing is not applicable to this article as no datasets were generated or analyzed during the current study. The corresponding author Juan Ambrosioni should be contacted.

## Abbreviations

ALT: Alanine aminotransferase
AST: Aspartate aminotransferase
FDG-PET/TC: ^18^F-fluorodeoxyglucose-positron emission tomography in combination with computed tomography scanning
GT: Gamma glutamyl transferase
NR: Not reported
Phosph: Phosphatase
PT: Prothrombin time

## References

[1] Burza S, Croft SL, Boelaert M (2018). Leishmaniasis. Lancet.

[2] Horrillo L, San Martín JV, Molina L, Madroñal E, Matía B, Castro A (2015). Atypical presentation in adults in the largest community outbreak of leishmaniasis in Europe (Fuenlabra, Spain). Clin Microbiol Infect.

[3] Wang H, Naghavi M, Allen C, Barber RM, Bhutta ZA, Carter A (2016). Global, regional, and national life expectancy, all-cause mortality, and cause-specific mortality for 249 causes of death, 1980-2015: a systematic analysis for the global burden of disease study 2015. Lancet.

[4] Girbau A, Baliellas C, Castellote J, de la Banda E (2010). Acute hepatitis and fever. Enferm Infecc Microbiol Clin.

[5] Sagnelli C, Di Martino F, Coppola N, Crisci A, Sagnelli E (2012). Acute liver failure: a rare clinical presentation of visceral leishmaniasis. New Microbiol.

[6] Bassegoda O, Solé C, Diaz A (2019). An unusual cause of acute severe hepatitis. Gastroenterology.

[7] Baranwal AK, Mandal RN, Singh R (2007). Fulminant hepatic failure complicating visceral leishmaniasis in an apparently immunocompetent child. Indian J Pediatr.

[8] Khanlari B, Bodmer M, Terracciano L, Heim MH, Fluckiger U, Weisser M (2008). Hepatitis with fibrin-ring granulomas. Infection.

[9] El Hag IA, Hashim FA, el Toum IA, Homeida M, el Kalifa M, el Hassan AM (1994). Liver morphology and function in visceral leishmaniasis (Kala-azar). J Clin Pathol.

